# A comprehensive multi-task deep learning model for kidney cancer: histological subtyping, clinical staging, and anatomical complexity grading

**DOI:** 10.1007/s00330-026-12322-z

**Published:** 2026-01-28

**Authors:** Dongqin Lv, Renyi Liu, Xiaochun Wang, Tianli Liang, Ling Zhang, Weixiong Zeng, Zilong He, Limei Deng, Zhi Zhang, Genggeng Qin, Weiguo Chen

**Affiliations:** 1 Department of Radiology, NanFang Hospital, Southern Medical University, Guangzhou, China; 2 Department of Radiology, Zhongshan Hospital of Traditional Chinese Medicine, Zhongshan, China; 3 Medical Imaging Center, Ganzhou People’s Hospital, Ganzhou, China

**Keywords:** Artificial intelligence, Deep learning, Kidney neoplasms, Tomography, X-ray computed

## Abstract

**Objectives:**

To develop and validate a multi-task deep learning (MTDL) model using multiphase contrast-enhanced CT (CECT) for simultaneously assessing histological subtypes, clinical stages, and anatomical complexity grades of solid malignant renal tumors.

**Materials and methods:**

This two-center retrospective study included patients with solid malignant renal tumors and their preoperative kidney CECT images. A progressive layered extraction (PLE)-based MTDL model was trained and externally tested. Model performance was evaluated using the area under the receiver operating characteristic curve (AUC) and decision curve analysis (DCA), compared with the results of five radiologists.

**Results:**

Among 798 patients (mean age, 54 ± 12 years; 279 females; Center A: *n* = 620, Center B: *n* = 178), 597 (74.8%) had clear cell renal cell carcinomas (ccRCC), 150 (18.8%) were clinical staging III/IV, and 187 (23.4%) had high anatomical complexity. On the external test set, the MTDL model achieved AUCs of 0.89 (95% CI: 0.82, 0.94) for distinguishing ccRCC from non-ccRCC, 0.87 (95% CI: 0.81, 0.93) for clinical staging (I/II vs. III/IV), and 0.87 (95% CI: 0.82, 0.92) for anatomical complexity grading (low-intermediate vs. high). The MTDL model outperformed single-task deep learning (STDL) in clinical staging (AUC: 0.87 vs. 0.82, *p* = 0.022), showed higher net benefit on DCA, and demonstrated better diagnostic performance than junior radiologists in histological subtyping and clinical staging. Additionally, it used 68% less memory and was 60% faster than STDL models.

**Conclusion:**

The CECT-based MTDL model demonstrated robust performance in simultaneously predicting histological subtypes, clinical stages, and anatomical complexity grades of malignant renal tumors.

**Key Points:**

***Question*** *Accurate preoperative description of the histological subtyping, clinical staging, and anatomical complexity of malignant renal tumors is crucial for treatment decision-making*.

***Findings*** *By sharing features, the multi-task deep learning algorithm model enhances clinical staging performance and significantly improves computational efficiency in predicting all three tasks simultaneously*.

***Clinical relevance*** *The multi-task deep learning algorithm model enables rapid and accurate comprehensive preoperative evaluation of renal tumors, which assists surgeons in optimizing surgical plans and promotes the advancement of renal tumor management toward precision and efficiency*.

**Graphical Abstract:**

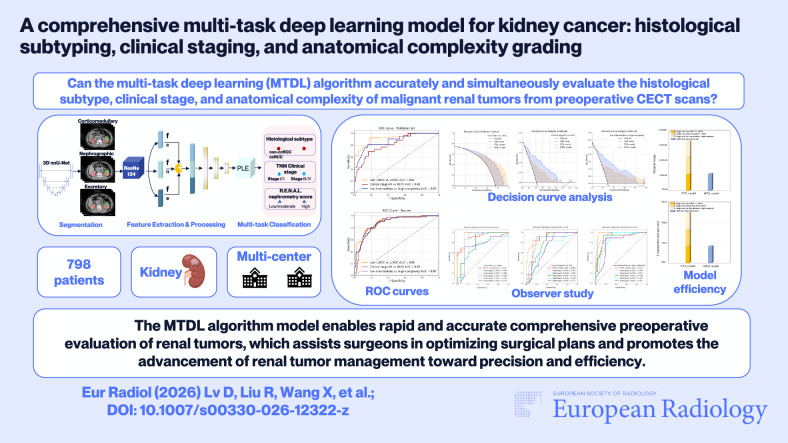

## Introduction

Renal tumors are one of the most common tumors in the urinary system, and their incidence is on the rise. The treatment options for malignant renal tumors include surgery, active surveillance, ablation, and neoadjuvant therapy, among others. The optimal approach should be determined based on a comprehensive tumor evaluation [1]. Histological subtype is considered an important factor, with clear cell renal cell carcinoma (ccRCC) being the most common subtype (75%–80% of cases) and associated with poor prognosis [2, 3]. Additionally, there are significant differences in drug regimen selection between advanced-stage non-ccRCC and ccRCC during neoadjuvant therapy [4]. Secondly, the TNM staging system can accurately stratify patients, further aiding in determining the optimal treatment approach. Localized renal cancer includes clinical stages I and II (T1–2N0M0), where partial nephrectomy (PN) is considered if technically feasible. In contrast, non-localized renal cancer (clinical stages III and IV) more often requires radical nephrectomy (RN) and comprehensive therapy [4]. The R.E.N.A.L. nephrometry score is a classic anatomical classification system that provides information on the technical complexity of tumor resection. Low-intermediate complexity tumors are more likely to be treated with PN and minimally invasive methods, while high-complexity tumors are more likely to undergo RN and open surgery [5, 6]. However, obtaining comprehensive preoperative supporting information is challenging. Pathological subtypes and TNM staging require invasive biopsy procedures. The R.E.N.A.L. nephrometry score relies on radiologists, who often work under time constraints, and the system is subject to scoring ambiguities and inter-observer variability. Therefore, an accurate and simultaneous preoperative assessment of these three elements—histological subtype, clinical stage, and anatomical complexity—is critical for formulating an optimal and individualized surgical treatment plan.

In recent years, deep learning (DL) has demonstrated promising potential in medical imaging [7]. However, most current research on renal image computation and analysis has focused on independently addressing different clinical tasks (i.e., constructing one model per task), overlooking meaningful connections between these tasks [8, 9]. Recently, multi-task deep learning (MTDL) has been explored to address this issue [10, 11], which can simultaneously handle multiple tasks with minimal supervision (i.e., one model solving multiple tasks), enhance learning efficiency through information sharing, and improve computational efficiency [12]. Among these advancements, the progressive layered extraction (PLE) significantly improves performance across varying task correlations and group scales [13]. It achieves this by separating shared and task-specific components and employing a progressive routing mechanism to gradually extract and isolate deeper semantic knowledge.

Currently, there are limited studies utilizing MTDL algorithms to simultaneously provide two or more types of renal tumor-related information, with most adopting simple frameworks that integrate multiple single-task models [14, 15]. Building on this, our study developed an MTDL model based on multiphase contrast-enhanced CT (CECT) and the PLE algorithm to simultaneously evaluate the histological subtype, clinical stage, and anatomical complexity grades of malignant renal tumors. Additionally, we constructed three single-task deep learning (STDL) models for each task and compared their performance with the MTDL model to analyze the clinical value of the MTDL approach.

## Materials and methods

The study was conducted in accordance with the Declaration of Helsinki and approved by the institutional ethics review boards of all participating hospitals (Approved number: NFEC-2024-285). Due to the retrospective nature of the datasets, the need for informed consent was waived; all datasets were independent of each other.

### Training and internal validation sets

To minimize selection bias, all consecutive patients with a single renal mass who underwent nephrectomy at Center A between January 2014 and June 2023 were included in the study. The inclusion criteria were as follows: (a) pathologically confirmed primary malignant renal tumors, (b) preoperative multiphase CECT scan (including corticomedullary, nephrographic, and excretory phases) with complete imaging of the kidneys and tumor. Exclusion criteria included: (a) cystic tumors (solid component < 25%) [16], (b) prior targeted therapy, immunotherapy, or chemoradiotherapy before CT examination, and (c) poor-quality CT images (e.g., metal artifacts or respiratory motion artifacts). Given the limited sample size of our study cohort (particularly for minority categories such as stage III/IV tumors), a completely randomized sampling approach was employed to assign patients to the training set and internal validation set at an 8:2 ratio, ensuring balanced distributions of all characteristics across both sets.

### External test set

**Fig. 1:**
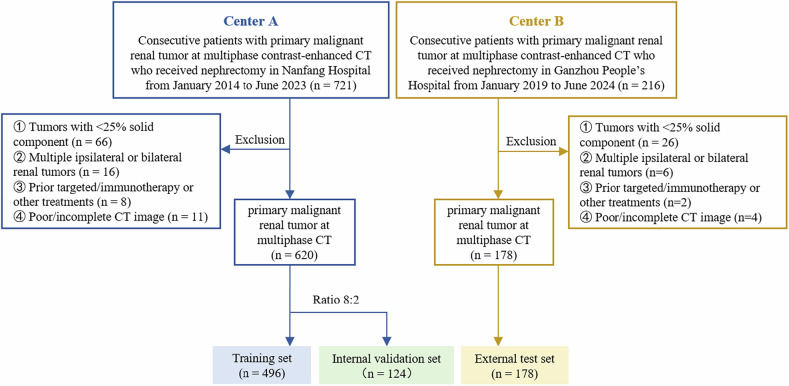
Flowchart of eligibility criteria and selection of the training, internal validation, and external test sets

Consecutive patients with a single renal mass who underwent nephrectomy at Center B between January 2019 and June 2024. The data from Center B served as the external test set, with identical inclusion and exclusion criteria to those of Center A. The workflow for patient inclusion and exclusion across all datasets is summarized in Fig. 1.

### Data preparation

The clinical data of all patients were obtained by reviewing the electronic medical record system and pathology system. Pathological subtype (non-ccRCC vs. ccRCC) diagnoses were verified according to the World Health Organization and International Society of Urological Pathology grading systems [17]. The TNM and clinical staging (I/II vs. III/IV) adhered to the 2023 European Association of Urology (EAU) guidelines on renal cell carcinoma [4]. Tumor anatomical complexity grades (low-intermediate vs. high complexity) were assessed by two experienced radiologists (G.Q., with 20 years of experience; W.C., with 30 years of experience) using the R.E.N.A.L. nephrometry score system, with detailed scoring criteria provided in Table S1. The inter-observer agreement for R.E.N.A.L. scores was assessed and is detailed in the supplementary material. Masses are classified as low complexity (R.E.N.A.L. score 4–6), moderate complexity (score 7–9), or high complexity (score 10–12). Compared to low-to-intermediate complexity tumors, high-complexity tumors more frequently require RN or open partial nephrectomy (PN) for preoperative decision-making and are associated with higher risks of postoperative complications [5, 18].

### Image segmentation

To ensure imaging quality control, a junior radiologist (D.L.) independently reviewed all CT scans following the inclusion and exclusion criteria. All CT images were anonymized and stored in NIFTI format. To obtain a tumor mask, we trained an nnU-Net automatic segmentation network on the KiTS23 dataset (supplementary materials). Data from both Center A and Center B served as independent external test sets for the segmentation model. The metrics, including the Dice coefficient, Relative Volume Error (RVE), and 95% Hausdorff Distance (HD95), were utilized to evaluate the performance of the segmentation model. The auto-segmented masks were jointly reviewed and manually corrected by two radiologists (T.L., 7 years of experience; X.W., 10 years of experience) using ITK-SNAP (version 3.8.0, http://www.itksnap.org). Additional details regarding CT scanning protocols and standardized segmentation procedures are provided in supplementary materials.

### Model development

**Fig. 2:**
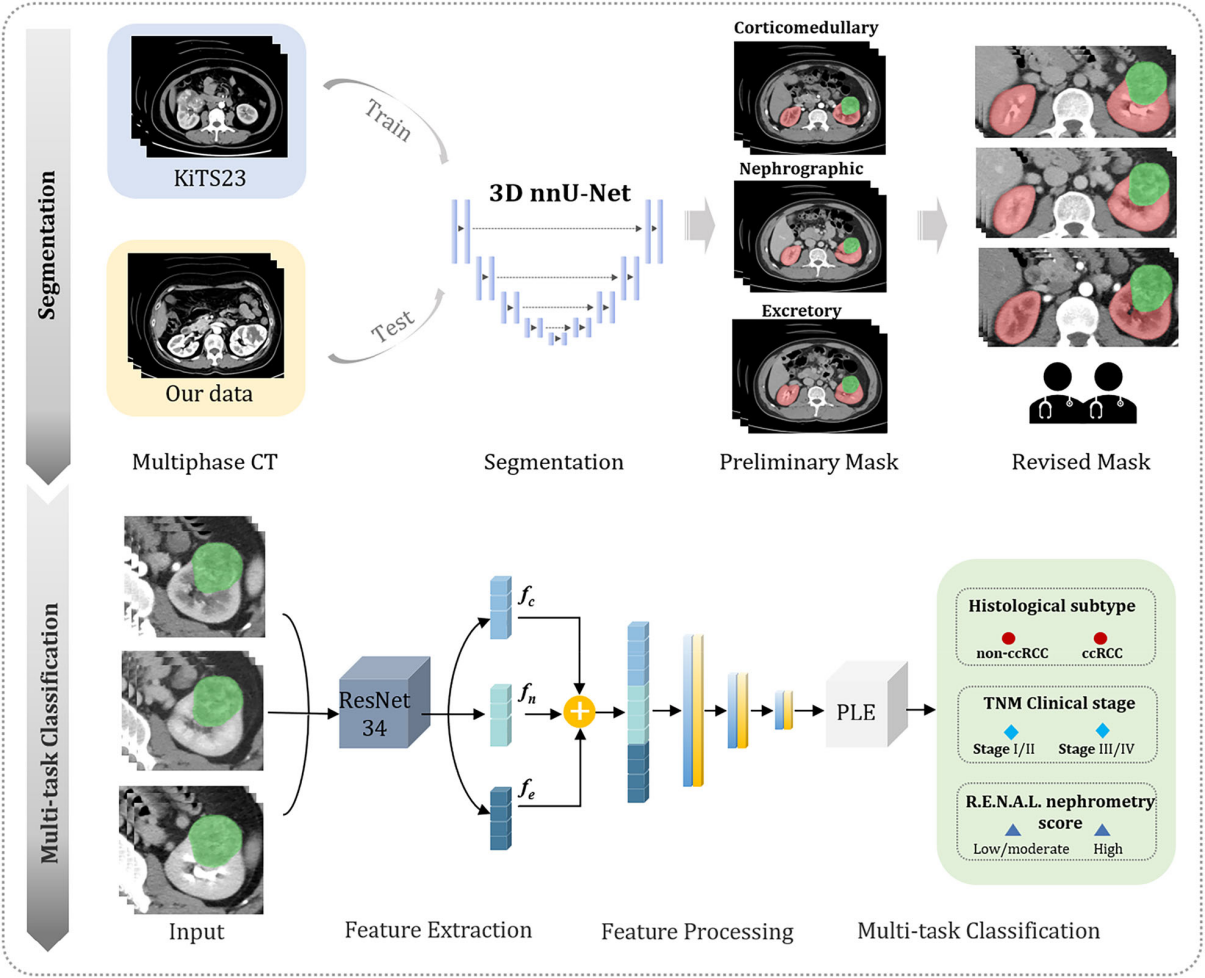
Schematic representation of the multi-task deep learning model development process. The *f*_c_, *f*_n_, and *f*_e_ represent the features extracted by the ResNet34 backbone from the corticomedullary phase, nephrographic phase, and excretory phase images, respectively. PLE, progressive layered extraction

A multi-task PLE model was developed to simultaneously classify: (a) non-ccRCC vs. ccRCC, (b) clinical stage I/II vs. stage III/IV, and (c) low-intermediate complexity vs. high complexity tumors. The Medical Open Network for Artificial Intelligence (MONAI, https://monai.io) DL framework was employed for CT image preprocessing and DataLoader construction. A 3D ResNet-34 backbone was then used to extract image features separately from the cortical phase, parenchymal phase, and excretory phase scans. These triphasic features were subsequently fused as input to the PLE module for simultaneous prediction across the three classification labels. Figure 2 illustrates the workflow of this study. For comparison, three STDL models were also constructed to predict the three labels, utilizing the same 3D ResNet-34 backbone for feature extraction and maintaining identical training parameters. All models were developed using the training set, with hyperparameter optimization performed on the internal validation set, followed by final evaluation on an independent external test set. Detailed network architecture is provided in the supplementary materials.

### Observer study

Five radiologists numbered from 1 to 5 (L.Z., Z.H., R.L., L.D., and Z.Z., with 12, 9, 6, 2, and 2 years of experience in abdominal imaging interpretation, respectively)—all of whom were independent from the reference standard annotation process—independently evaluated the external test set to assess the probability (0%–100%) of each case being classified as ccRCC, clinical stage III/IV, and high complexity according to the R.E.N.A.L. nephrometry score. To ensure data confidentiality, all cases underwent randomization and anonymization prior to evaluation. Before the assessment, all observers completed a standardized training program that included analysis of characteristic imaging findings in representative cases, interpretation of clinical staging criteria based on the 2023 EAU guidelines and a comprehensive review of the R.E.N.A.L. nephrometry score system literature [4, 5]. The diagnostic performance of the MTDL model was subsequently compared against the radiologists’ evaluations.

### Statistical analysis

The diagnostic performance of both the MTDL and STDL models was evaluated using metrics with 95% confidence intervals (CI), including sensitivity, specificity, accuracy, and the area under the receiver operating characteristic curve (AUC). The optimal threshold for the models was determined by maximizing the Youden index. Correspondingly, the performance of five radiologists was evaluated. The MTDL model’s AUC values were compared against both the STDL model and radiologists using the DeLong test. Decision curve analysis (DCA) was employed to assess the clinical utility of the models. Computation time and memory usage were employed to compare the efficiency between MTDL and STDL models. Continuous variables were presented as mean ± standard deviation (SD), while categorical variables were expressed as counts and percentages. The comparison of baseline characteristics among the three datasets was performed using one-way ANOVA and the chi-square test. All statistical analyses and experiments were conducted using Python (version 3.8.0, www.python.org) and R (version 4.4.2, www.r-project.org). Two-sided *p* < 0.05 was considered to indicate a statistically significant difference. To compare the overall performance between the MTDL and STDL models across the three tasks, the Bonferroni correction was applied for multiple comparisons of *P* values.

## Result

### Study population

The baseline characteristics of the datasets are summarized in Table 1 and S3. After applying the inclusion and exclusion criteria (Fig. 1), a total of 798 patients with malignant renal tumors (mean age, 54 years ± 12 (SD); 279 females) were included. A total of 597 (74.8%) tumors were ccRCC, 150 (18.8%) were clinical staging III/IV, and 187 (23.4%) were high anatomical complexity grade. Center A cohort comprised 620 patients (mean age, 53 years ± 13, 218 females), including 463 (74.7%) ccRCC and 157 (25.3%) non-ccRCC; 507 (81.8%) in clinical stage I/II, and 113 (18.2%) in clinical stage III/IV; according to R.E.N.A.L. nephrometry scores, 484 (78.1%) with low-intermediate complexity, and 136 (21.9%) with high complexity. Center B cohort included 178 patients (mean age 58 years ± 11, 61 females), consisting of 134 (75.3%) ccRCC, 141 (79.2%) in clinical stage I/II, and 127 (71.3%) with low-to-intermediate complexity. Age distribution differed significantly between the two centers (*p* < 0.001). The inter-observer agreement for R.E.N.A.L. scores was good, as shown in Table S2.

**Table 1:** Baseline characteristics of the datasets

	Center A (*n* = 620)		Center B	
Characteristic	Training set (*n* = 496)	Validation set (*n* = 124)	Test set (*n* = 178)	*p*-value
Mean age (years)	53 ± 12	54 ± 13	58 ± 11	< 0.001
Sex				0.935
Female	173 (34.9)	45 (36.3)	61 (34.3)	
Male	323 (65.1)	79 (63.7)	117 (65.7)	
BMI (kg/m²)	24.7 ± 9.9	24.6 ± 3.7	23.6 ± 3.5	0.316
Maximum diameter	5.3 ± 2.8	5.4 ± 2.7	5.5 ± 3.1	0.693
Pathology type				0.477
Clear cell RCC	361 (72.8)	102 (82.3)	134 (75.3)	
Papillary RCC	35 (7.0)	5 (4.0)	10 (5.6)	
Chromophobe RCC	55 (11.1)	10 (8.1)	16 (9.0)	
Other malignant	45 (9.1)	7 (5.6)	18 (10.1)	
Task 1				0.093
Clear cell RCC	361 (72.8)	102 (82.3)	134 (75.3)	
non-Clear cell RCC	135 (27.2)	22 (17.7)	44 (24.7)	
Task 2				0.507
Stage I/II	409 (82.5)	98 (79.0)	141 (79.2)	
Stage III/IV	87 (17.5)	26 (21.0)	37 (20.8)	
Task 3				0.060
Complexity low-intermediate	381 (76.8)	103 (83.1)	127 (71.3)	
Complexity high	115 (23.2)	21 (16.9)	51 (28.7)	

Note: Continuous variables were presented as mean ± standard deviation (SD), while categorical variables were expressed as counts and percentages. The *p*-value indicates the statistical differences between the training set, validation set, and test set.Task 1 is histological subtypes, Task 2 is clinical stages, and Task 3 is anatomical complexity grades based on R.E.N.A.L. nephrometry scores

*BMI* body mass index, *RCC* renal cell carcinoma

### Segmentation performance of the nnU-Net model

This model demonstrated good segmentation performance across all CT phases of Center A’s data, with Dice coefficients of 0.955–0.961, RVE of 4.7%–5.7%, and HD95 of 1.51–1.72 mm. For Center B, the results showed Dice coefficients of 0.901–0.909, RVE of 15.7%–18.9%, and HD95 of 2.16–2.70 mm. Detailed results are provided in Table S5.

### Diagnostic performance of the MTDL algorithm

The diagnostic performance of the MTDL algorithm model for three tasks—differentiating non-ccRCC from ccRCC, clinical stage I/II from III/IV, and low-intermediate from high complexity R.E.N.A.L. nephrometry scores—in both the internal validation set and external test set is presented in Table 2 and S7. In the external test set, the algorithm achieved a sensitivity of 90% (95% CI: 84, 95) and specificity of 73% (95% CI: 59, 86) with an AUC of 0.89 (95% CI: 0.82, 0.94) for distinguishing non-ccRCC from ccRCC. For clinical staging between I/II and III/IV, it showed a sensitivity of 78% (95% CI: 65, 91), specificity of 84% (95% CI: 78, 90), and AUC of 0.87 (95% CI: 0.81, 0.93). For classifying low-intermediate vs. high complexity, it demonstrated a sensitivity of 88% (95% CI: 80, 96), specificity of 74% (95% CI: 66, 82), and an AUC of 0.87 (95% CI: 0.82, 0.92).

**Table 2:** Performance of the multi-task and single-task deep learning algorithms on each task

Metric	Task 1			Task 2			Task 3		
	MTDL	STDL	*p*-value (adjusted *p*)	MTDL	STDL	*p*-value (adjusted *p*)	MTDL	STDL	*p*-value (adjusted *p*)
Validation set (*n* = 124)									
Sensitivity (%)^a^	91 (93/102)	88 (90/102)	0.450	69 (18/26)	88 (23/26)	0.074	81 (17/21)	90 (19/21)	0.480
Specificity (%)^a^	82 (18/22)	91 (20/22)	0.617	84 (82/98)	70 (69/98)	**0.012**	84 (87/103)	81 (83/103)	0.423
Accuracy (%)^a^	90 (111/124)	89 (110/124)	1.000	81 (100/124)	74 (92/124)	0.186	84 (104/124)	82 (102/124)	0.803
AUC^b^	0.92 (0.86, 0.97)	0.94 (0.90, 0.97)	0.489 (1.000)	0.82 (0.72, 0.90)	0.84 (0.74, 0.93)	0.607 (1.000)	0.87 (0.77, 0.96)	0.92 (0.86, 0.97)	0.188 (0.564)
Test set (*n* = 178)									
Sensitivity (%)^a^	90 (121/134)	85 (114/134)	0.121	78 (29/37)	81 (30/37)	1.000	88 (45/51)	86 (44/51)	1.000
Specificity (%)^a^	73 (32/44)	75 (33/44)	1.000	84 (119/141)	71 (100/141)	**< 0.001**	74 (94/127)	79 (100/127)	0.264
Accuracy (%)^a^	86 (153/178)	83 (147/178)	0.307	83 (148/178)	73 (130/178)	**< 0.001**	78 (139/178)	81 (144/178)	0.424
AUC^b^	0.89 (0.82, 0.94)	0.88 (0.82, 0.94)	0.945 (1.000)	0.87 (0.81, 0.93)	0.82 (0.75, 0.88)	**0.022** (0.066)	0.87 (0.82, 0.92)	0.88 (0.83, 0.93)	0.577 (1.000)

Note: Sensitivity, specificity, and accuracy are reported as percentages, with proportions of patients (numerator/denominator) in parentheses. Sensitivity = $\frac{{TP}}{{TP}+{FN}}\times 100 \%$, Specificity = $\frac{{TN}}{{TN}+{FP}}\times 100 \%$, Accuracy = $\frac{{TP}+{TN}}{{TP}+{TN}+{FP}+{FN}}\times 100 \%$, Task 1 is non-ccRCC vs. ccRCC, Task 2 is clinical stage I/II vs. III/IV, Task 3 is low-intermediate vs. high complexity

*TN* true negatives, *TP* true positives, *FN* false negatives, *FP* false positives, *MTDL* multi-task deep learning, *STDL* single-task deep learning, *AUC* area under the receiver operating characteristic curve

^a^ *p*-value was calculated with the McNemar’s Chi-squared test

^b^ *p*-value was calculated with the DeLong test, and data in parentheses are 95% CIs. To compare overall performance across the three tasks, the adjusted *p*-value was obtained using the Bonferroni correction

Bold values represent results with statistical significance (*p* < 0.05)

### Comparison with the STDL algorithm

For differentiating non-ccRCC from ccRCC, the performance comparison between the MTDL and STDL algorithm models demonstrated comparable AUC values (internal validation set: 0.92 [95% CI: 0.86, 0.97] vs. 0.94 [95% CI: 0.90, 0.97], *p* = 0.489; external test set: 0.89 [95% CI: 0.82, 0.94] vs. 0.88 [95% CI: 0.82, 0.94], *p* = 0.954). DCA revealed higher net benefits for MTDL at thresholds 0–0.9 in validation and test sets. The STDL model performed better at thresholds > 0.9, but its net benefit was lower than the treat-all strategy at thresholds between 0 and 0.4.

For the clinical stage task, the MTDL and STDL models showed comparable AUC in the internal validation set (0.82 [95% CI: 0.72, 0.90] vs. 0.84 [95% CI: 0.74, 0.93], *p* = 0.607), while MTDL demonstrated superior specificity (84% [95% CI: 76, 90] vs. 70% [95% CI: 61, 79], *p* = 0.012). In the external test set, MTDL significantly outperformed STDL across all metrics: specificity (84% [95% CI: 78, 90] vs. 71% [95% CI: 64, 78], *p* < 0.001), accuracy (83% [95% CI: 78, 88] vs. 73% [95% CI: 66, 79], *p* < 0.001), and AUC (0.87 [95% CI: 0.81, 0.93] vs. 0.82 [95% CI: 0.75, 0.88], *p* = 0.022). DCA showed MTDL had greater net benefit than STDL at thresholds of 0–0.5 in the test set.

**Fig. 3:**
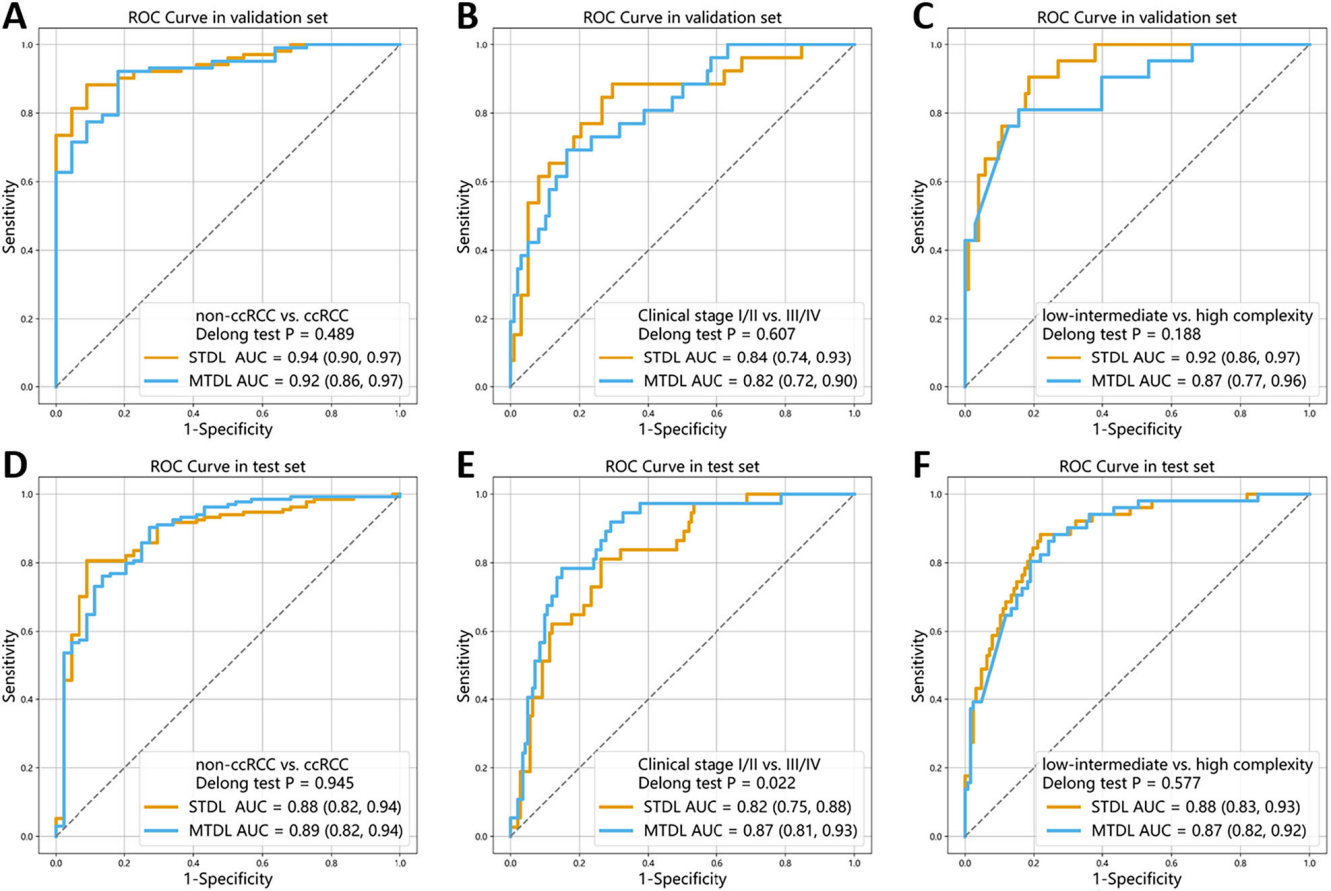
Receiver operating characteristic curves of the MTDL and STDL algorithms in identification of (**A**, **D**) histological subtypes, (**B**, **E**) clinical stages, and (**C**, **F**) anatomical complexity grades of renal tumors in the (**A**–**C**) internal validation set, and (**D**–**F**) external test set

**Fig. 4:**
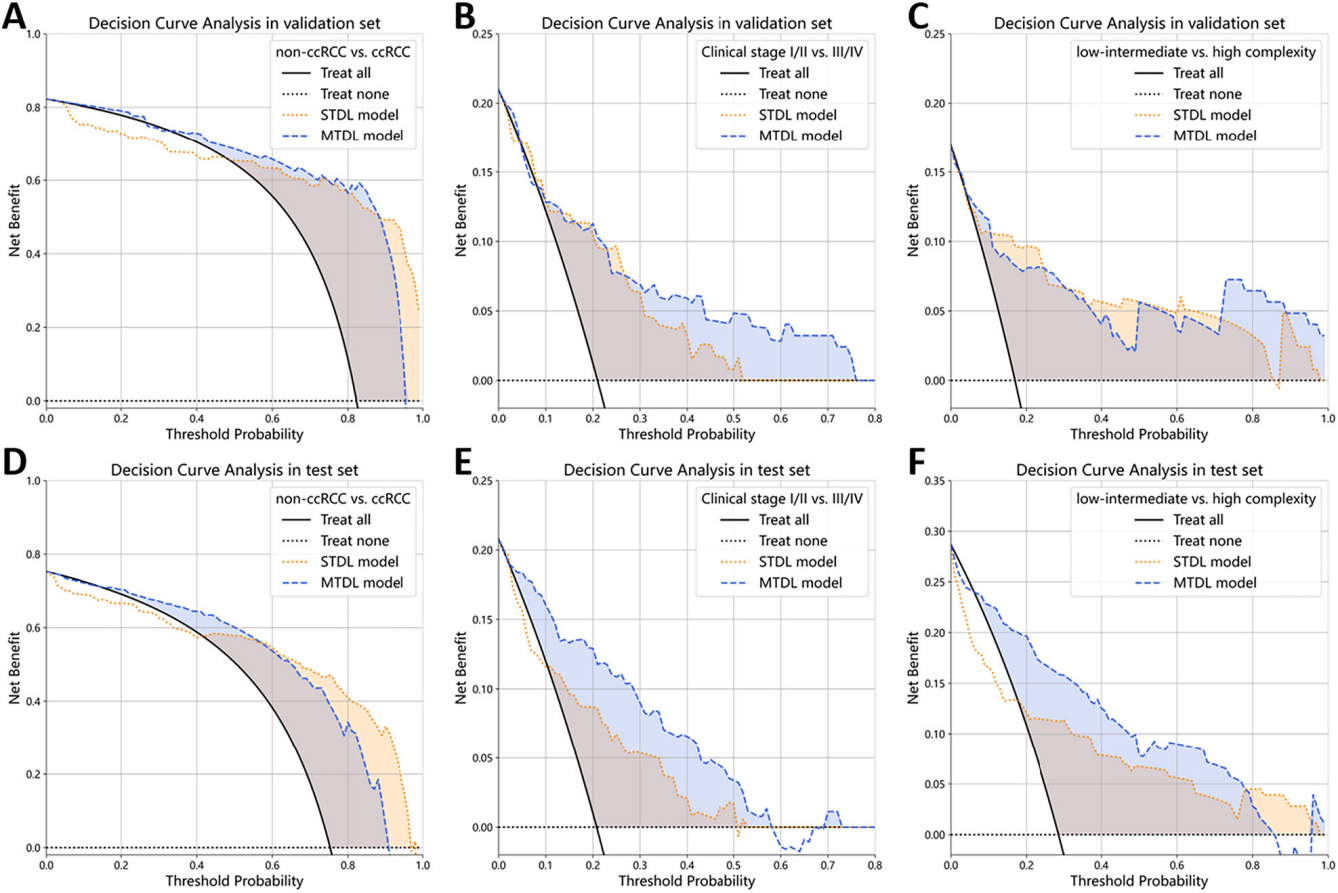
Decision curve analysis of the MTDL and STDL algorithms in identification of (**A**, **D**) histological subtypes, (**B**, **E**) clinical stages, and (**C**, **F**) anatomical complexity grades of renal tumors in the (**A**–**C**) internal validation set, and (**D**–**F**) external test set. The *x*-axis shows the decision threshold probability (0%–100%), the minimum predicted probability at which a clinician would intervene. The *y*-axis indicates the net benefit. The black solid and dashed lines represent the “treat-all” and “treat-none” reference strategies, respectively. The yellow and blue dashed curves correspond to the study models. A model provides clinical value when its curve lies above both reference lines; higher curves indicate greater net benefit

For the R.E.N.A.L. nephrometry score task, no significant differences in AUC were observed between the MTDL and STDL algorithm models (internal validation set: 0.87 [95% CI: 0.77, 0.96] vs. 0.92 [95% CI: 0.86, 0.97], *p* = 0.188; external test set: 0.87 [95% CI: 0.82, 0.92] vs. 0.88 [95% CI: 0.83, 0.93], *p* = 0.577). Besides, DCA showed MTDL had a greater net benefit than STDL at thresholds of 0–0.8 in the test set. The comparative diagnostic performance of MTDL and STDL models is presented in Table 2 and Figs. 3 and 4.

In comparing the overall performance across the three tasks of histological subtyping, clinical staging, and anatomical complexity grading, no statistically significant differences in AUC were observed between the MTDL and STDL models in either the internal validation set (adjusted *p*: 1.000, 1.000, 0.564) or the external test set (adjusted *p*: 1.000, 0.066, 1.000).

### Model efficiency comparison

On the external test set, the three STDL models for histological subtyping, clinical staging, and R.E.N.A.L. nephrometry score collectively required 1065 megabytes of memory. In contrast, the MTDL model needed 335 megabytes of memory while simultaneously processing all three tasks, achieving an approximate 68% reduction in memory usage (Fig. S1). Furthermore, the MTDL model demonstrated significantly faster prediction times per case (19.9 ms vs. 50.6 ms for the STDL model), corresponding to a 60% increase in speed.

### Comparison with radiologists’ assessment

**Fig. 5:**
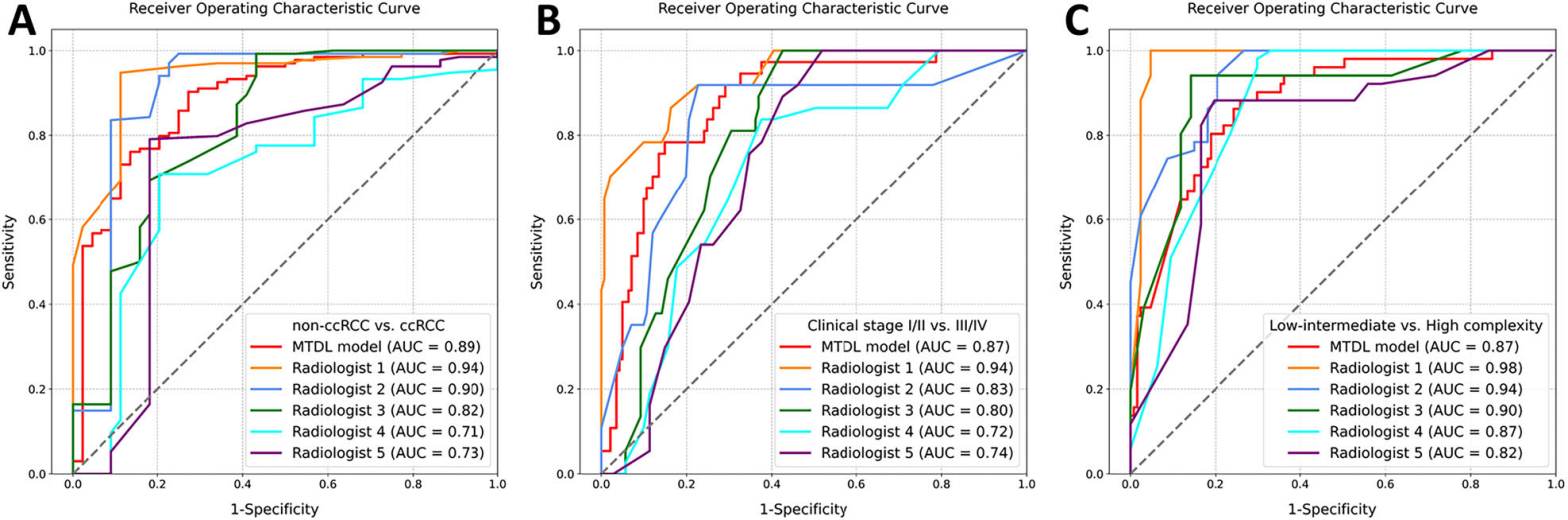
Receiver operating characteristic curves of the deep learning algorithm and clinicians in the identification of **A** histological subtypes, **B** clinical stages, and **C** anatomical complexity grades of renal tumors

**Fig. 6:**
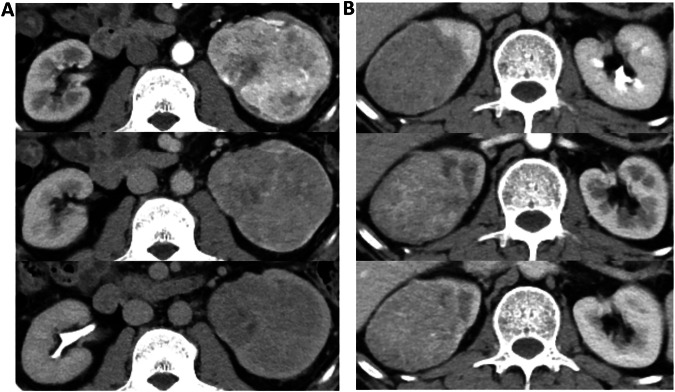
Two examples from the test set are shown. **A** A 51-year-old male patient with a clinical stage II and highly complex clear cell renal cell carcinoma (ccRCC) in the left kidney. The lesion exhibited indistinct margins and was closely related to the collecting system. This case was misclassified as a high clinical stage (III/IV) tumor by the single-task deep learning (STDL) model but was correctly classified as a low clinical stage tumor by the multi-task deep learning (MTDL) model. **B** A 47-year-old male patient with a clinical stage II ccRCC in the right kidney, anatomically classified as highly complex. The tumor showed indistinct margins and slightly lower enhancement than the renal cortex in the arterial phase. This case was misclassified as clinical stage III ccRCC by junior radiologist 5 and as clinical stage III non-ccRCC by junior radiologist 4, but was correctly classified by the MTDL model as ccRCC, low clinical stage (I/II), and highly complex

Table 3 and Fig. 5 show radiologists’ diagnostic performance. For the comparison of individual tasks, the MTDL model had higher AUCs than junior radiologists 4, 5 in histological subtyping (0.89 [95% CI: 0.82, 0.94] vs. 0.71 [95% CI: 0.62, 0.80], *p* = 0.001; 0.73 [95% CI: 0.62, 0.83], *p* = 0.005). In clinical staging, the MTDL model outperformed radiologists 3–5(0.87 [95% CI: 0.81, 0.93] vs. 0.80 [95% CI: 0.74, 0.86], *p* = 0.041; 0.72 [95% CI: 0.64, 0.80], *p* = 0.001; 0.74 [95% CI: 0.67, 0.81], *p* = 0.002). For anatomical complexity, senior radiologists 1, 2 outperformed MTDL (0.87 [95% CI: 0.82, 0.92] vs. 0.98 [95% CI: 0.96, 1.00], *p* < 0.001; 0.94 [95% CI: 0.91, 0.97], *p* = 0.024). Regarding the overall performance across the three tasks, in both histological subtyping and clinical staging tasks, the MTDL model performed better than junior radiologists 4 (adjusted *p*: 0.004, 0.001) and 5 (adjusted *p*: 0.014, 0.007) (Fig. 6).

**Table 3:** Performance of the multi-task deep learning algorithm and radiologists in the test set

Variable	Task 1				Task 2				Task 3			
	Sen/Spe (%)	AUC	*p-*value^a^	Adjusted *p-*value^b^	Sen/Spe (%)	AUC	*p-*value^a^	Adjusted *p-*value^b^	Sen/Spe (%)	AUC	*p-*value^a^	Adjusted *p*-value^b^
MTDL model	90 (85, 94) /73 (59, 86)	0.89 (0.82, 0.94)			78 (64, 90) /84 (78, 90)	0.87 (0.81, 0.93)			88 (78, 96) /74 (67, 82)	0.87 (0.82, 0.92)		
Radiologist 1	95 (90, 98)/89 (78, 97)	0.94 (0.89, 0.97)	0.137	0.411	86 (74, 97)/84 (77, 89)	0.94 (0.90, 0.97)	**0.021**	0.060	100 (100, 100)/95 (92, 98)	0.98 (0.96, 1.00)	**< 0.001**	**< 0.001**
Radiologist 2	84 (77, 90)/91 (80, 98)	0.90 (0.81, 0.96)	0.773	1.000	92 (83, 100)/77(70, 84)	0.83 (0.74, 0.90)	0.263	0.788	94 (87, 100)/80 (73, 86)	0.94 (0.91, 0.97)	**0.024**	0.071
Radiologist 3	99 (98, 100)/57 (41, 71)	0.82 (0.73, 0.90)	0.200	0.600	89 (79, 97) /63 (56, 71)	0.80 (0.74, 0.86)	**0.041**	0.122	94 (87, 100)/86 (80, 91)	0.90 (0.84, 0.95)	0.497	1.000
Radiologist 4	71 (63, 79)/80 (67, 91)	0.71 (0.62, 0.80)	**0.001**	**0.004**	84 (70, 94) /62 (54, 70)	0.72 (0.64, 0.80)	**0.001**	**0.002**	98 (93, 100)/70 (62, 78)	0.87 (0.82, 0.92)	0.926	1.000
Radiologist 5	79 (71, 86)/82 (70, 93)	0.73 (0.62, 0.83)	**0.005**	**0.014**	89 (77, 97)/57 (49, 65)	0.74 (0.67, 0.81)	**0.002**	**0.007**	88 (79, 96)/80 (73, 87)	0.82 (0.75, 0.89)	0.156	0.468

Note: Unless otherwise specified, values in parentheses represent the 95% confidence interval. Task 1 is non-ccRCC vs. ccRCC, Task 2 is clinical stage I/II vs. III/IV, and Task 3 is low-intermediate vs. high complexity

*Sen* sensitivity. *Spe* specificity. *MTDL* multi-task deep learning. *AUC* area under the receiver operating characteristic curve

^a^*p*-values represent comparisons between the MTDL algorithm model and each radiologist for each task

^b^To compare overall performance across the three tasks, the adjusted *p*-value was obtained using the Bonferroni correction

Bold values represent results with statistical significance (*p* < 0.05)

## Discussion

Preoperative comprehensive evaluation of malignant renal tumors remains a clinical challenge. In this study, we developed an MTDL model using multiphase contrast-enhanced CT (CECT) to simultaneously predict histological subtype, clinical stage, and anatomical complexity. This model provides surgeons with integrated preoperative insights to guide surgical planning and may serve as a non-invasive adjunct for patients undergoing biopsy. In an independent external test cohort, the MTDL model demonstrated robust diagnostic performance, achieving an AUC of 0.89 (95% CI: 0.82, 0.94) for discriminating non-ccRCC from ccRCC, 0.87 (95% CI: 0.81, 0.93) for predicting clinical stage I/II vs. III/IV, and 0.87 (95% CI: 0.82, 0.92) for predicting low-intermediate (R.E.N.A.L. score 4–9) vs. high complexity (score 10–12) tumors.

Deep learning, a specialized field within machine learning, fundamentally involves automatically learning features from data and accomplishing tasks through multi-layer neural networks. Currently, it is playing a transformative role in renal imaging [9]. Xiong et al [19] developed two convolutional neural network (CNN) models based on a large-sample, multicenter cohort for diagnosing renal tumor malignancy and distinguishing aggressive from indolent tumors, respectively. Both models demonstrated strong performance, with AUC values of 0.871 and 0.783. Moreover, these DL models outperformed corresponding radiomics models, which often require complex manual feature extraction and feature engineering. When a model focuses solely on solving a single clinical problem, it is referred to as an STDL model. In such models, all network structures and parameter optimization are dedicated exclusively to one task, offering the advantages of simplicity and targeted parameter tuning. However, they suffer from low data utilization efficiency and are prone to overfitting.

Unlike conventional STDL, MTDL constructs a single model to simultaneously train two or more tasks, requiring only a single pass of data loading and computation, thereby demonstrating high efficiency. Moreover, by sharing features and parameters across tasks, the model learns representations that must adapt to multiple objectives, which helps reduce task-specific overfitting and enhances generalization performance [20, 21]. MTDL enhances performance across related tasks by simultaneously optimizing multiple loss functions. Specifically, if *L*ᵢ represents the loss function for task Tᵢ, the model’s total loss function is defined as: ${{{{\rm{L}}}}}_{{{{\rm{MTDL}}}}}={\sum }_{i=1}^{m}{w}_{{{{\rm{i}}}}}\bullet {L}_{{{{\rm{i}}}}}$, where *w*_i_ denotes a weighting term that balances the individual losses across tasks [10]. This approach accounts for inter-task variability. However, a challenge involves balancing loss functions among concurrent tasks, where improper weight settings can easily lead to model bias toward a specific task. In this study, we employed equal weighting for the loss summation across the three tasks (i.e., *w*ᵢ = 1:1:1 for all tasks), which avoided overfitting to the validation set performance that might result from complex weight search procedures, thereby ensuring the reproducibility of the results. By comparing the performance of the equal-weight model with that of STDL models, we could determine whether positive mutual enhancement or negative interference exists between the different tasks [11].

Previous limited studies have employed multi-task networks for renal tumor analysis. Ruan et al [22] proposed a multi-branch feature-sharing generative adversarial network that employs joint learning and adversarial learning to accurately segment and quantify renal tumors. Their method effectively leveraged both commonalities and differences between the two related tasks, achieving a tumor segmentation accuracy of 95.7% and a diameter correlation coefficient of 0.904. Zeng et al [23] developed a 2D DL convolutional network based on an unsupervised domain adaptation mechanism for automated generation of similar kidney images and segmentation of renal anatomical structures, which outperformed supervised learning-based kidney segmentation methods. These studies leverage the mutual enhancement effect of related tasks to improve the model’s segmentation performance. Considering these advantages, we hypothesized that the three tasks—histological subtyping, clinical staging, and anatomical complexity assessment of renal tumors—might share certain common features. By integrating these clinically relevant tasks, we established an MTDL model based on the ResNet-34 backbone and evaluated it on an independent test set.

In recent years, most artificial intelligence studies on renal tumor imaging have focused on developing single-task networks to address specific clinical problems. Han et al [24] employed a DL framework based on CECT images to differentiate three major renal cell carcinoma (RCC) subtypes: clear cell RCC (ccRCC), papillary RCC, and chromophobe RCC. Their model achieved 65% sensitivity and an AUC of 0.936 in distinguishing non-ccRCC from ccRCC. However, the generalizability and robustness remain uncertain due to the limited sample size (*n* = 169) and lack of external validation. Uhm et al [25] developed an end-to-end DL model for differential diagnosis of five major renal tumor subtypes. The model showed an AUC of 0.864 (95% CI: 0.696, 0.989) in the internal validation set and 0.917 (95% CI: 0.805, 0.997) in the external test set for non-ccRCC vs. ccRCC classification. Although this study improved upon previous work by increasing sample size and incorporating external validation, it essentially decomposed the histological subtyping task into five independent binary classification problems. Wu et al [15] proposed a CT radiomics-based multi-task framework to assess tumor malignancy, histological subtype, pathological stage, Fuhrman grade, and Ki-67 index. In their external cohort, the optimal model achieved AUCs of 0.83 (95% CI: 0.76, 0.89) for non-ccRCC/ccRCC discrimination and 0.85 (95% CI: 0.79, 0.92) for T1/T2 vs. T3/T4 staging. Similarly, Yang et al [26] developed an AI framework utilizing a multi-scale feature fusion strategy, which incorporated machine learning models for classifying renal tumor pathological grades and Ki-67 indices, achieving strong overall performance. While innovative, these approaches essentially stacked multiple single-task networks within one framework, resulting in low data utilization efficiency. In contrast, our study utilized an MTDL network model while achieving overall predictive performance across the three tasks comparable to that of STDL. Additionally, Heller et al [27] utilized a CT-based deep neural network to automatically segment kidneys and tumors while estimating R.E.N.A.L. nephrometry scores. Their results demonstrated moderate agreement between model-generated and expert radiologist scores (concordance coefficient = 0.6, *p* < 0.0001). In contrast, our study simplifies this workflow by focusing on the clinically critical distinction between low-intermediate vs. high complexity tumors, demonstrating robust performance that provides surgeons with intuitive decision support.

The interconnected tasks in MTDL networks can mutually enhance or diminish performance. When one task contains information beneficial to another, it improves performance on the latter; conversely, it may cause interference [28–30]. In this study, to accurately and fairly evaluate the interactions (synergistic or interfering) between different tasks in the MTDL model, we constructed three STDL models employing identical pre-trained backbones and training parameters. The MTDL model demonstrated superior diagnostic performance for clinical staging compared to STDL in the external test set (AUC: 0.87 vs. 0.82). Decision curve analysis (DCA) revealed that the MTDL model provided better clinical utility than the STDL model across a wider threshold probability range (0.1–0.5). Correlation analysis revealed statistically significant interrelationships among the three clinical prediction tasks, particularly between staging and complexity (Table S8). Key components of the R.E.N.A.L. nephrometry score—including larger tumor diameter, closer proximity to the collecting system/renal sinus, and lower exophytic rate—show significant associations with advanced clinical stage. Demirjian et al [31] developed a CT-based radiomics machine learning model for distinguishing stage I/II from III/IV, reporting a maximum AUC of 0.80 for their full feature model—comparable to our STDL performance and lower than that of the MTDL model. This advantage was also evident in the Observers' study, where the MTDL algorithm outperformed junior radiologists in clinical staging assessment. Moreover, our MTDL model surpassed junior radiologists across most tasks (particularly for histological subtyping and clinical staging) while achieving comparable performance to senior radiologists. Notably, the performance of the MTDL model was comparable to that of STDL models in histological subtyping and anatomical complexity classification, which may be because these tasks experienced limited positive transfer or, at least, did not gain additional benefits from shared representation. Approximately 25%–30% of ccRCC patients present with metastasis at initial diagnosis, and ccRCC exhibits more aggressive behavior with poorer prognosis [32, 33]. However, in this study, histological subtyping showed a weak correlation with the other tasks, likely due to the diverse subtypes included within the non-ccRCC category, which may have obscured distinct associated imaging features. Moreover, since the losses of the three tasks can differ significantly in scale, a fixed 1:1:1 weighting risks allowing one task (e.g., clinical staging) to dominate the gradient updates, suppressing the learning of the remaining tasks.

On the other hand, the multi-task network architecture allows parameter sharing across tasks, thereby reducing memory footprint and minimizing redundant computations during inference to improve processing speed—a frequently overlooked yet critical advantage. This is particularly significant in healthcare settings, where data management poses major challenges for medical institutions, with imaging data accounting for approximately 90% of all medical data [34]. Moreover, the recent rapid expansion of intelligent medical devices and AI applications—such as InferRead CT Lung for pulmonary nodule detection (https://www.infervision.com), Viz 3D CTA for neurovascular analysis (https://www.viz.ai/), and CoronaryDoc for cardiovascular assessment (https://www.shukun.com) in medical imaging—highlights the critical need for computational efficiency. From 2019 to mid-2023, the Food and Drug Administration approved nearly 700 AI medical devices, with over 75% of them used in radiology [9]. Under conventional data management conditions, the high-efficiency AI models could alleviate resource constraints in clinical environments [10, 35]. Compared to STDL models, our MTDL model achieved a 68% reduction in memory consumption (335 megabytes vs. 1065 megabytes) when simultaneously processing all three tasks, along with a 60% shorter prediction time per case (19.9 ms vs. 50.6 ms). When diagnostic capabilities are comparable, this efficiency enhancement makes the MTDL algorithm more amenable to integration into clinical workflows than the STDL algorithm.

Our study has several limitations. First, the model was developed based on a surgical cohort, which may limit its generalizability to early-stage or non-surgical patients. Second, since this prediction model was developed based on retrospective medical records and the datasets did not incorporate the temporal splitting strategy, prospective cohort validation is particularly required in future studies. Third, regarding the limited sample size of advanced-stage (III/IV) and high-complexity tumors, as well as the potential risk of false positive results, although we employed an independent external validation design, the external cohort was derived from a single medical center with a limited sample size. Therefore, further validation across additional medical centers remains necessary in future studies. Furthermore, the model’s generalizability across diverse ethnic populations requires further validation through international multicenter studies. Fourth, equal task weighting in the MTDL model provided a stable baseline but ignored differences in difficulty and class imbalance. Future work should explore refined weighting to improve performance. Finally, the predictive model cannot completely replace invasive pathological examinations. While it can assist physicians in preliminarily assessing the histological subtype, clinical stage, and anatomical complexity preoperatively to guide surgical planning, it remains unclear whether this approach can ultimately improve patient outcomes—this requires long-term follow-up data and prospective studies for verification.

In conclusion, we have developed a non-invasive MTDL model based on preoperative multiphase contrast-enhanced CT that can simultaneously and accurately predict histological subtypes, clinical staging, and anatomical complexity. Furthermore, the shared design of the MTDL network enables improved performance in clinical staging without compromising histological or anatomical complexity classification. This MTDL algorithm can assist surgeons in optimizing surgical planning and contribute to more precise and efficient management of renal tumors.

## Supplementary information

Supplementary Material

## Abbreviations

AUC: Area under the receiver operating characteristic curve
ccRCC: Clear cell renal cell carcinoma
CECT: Contrast-enhanced CT
CI: Confidence interval
DCA: Decision curve analysis
DL: Deep learning
EAU: European Association of Urology
KiTS23: The 2023 Kidney and Kidney Tumor Segmentation Challenge
MTDL: Multi-task deep learning
PLE: Progressive layered extraction
PN: Partial nephrectomy
RN: Radical nephrectomy
SD: Standard deviation
STDL: Single-task deep learning
