# Supplementary material for: A comprehensive multi-task deep learning model for kidney cancer: histological subtyping, clinical staging, and anatomical complexity grading
Source: Eur Radiol. 2026 Jan 28;36(6):4513–26. doi: 10.1007/s00330-026-12322-z (PMC13212381; doi:10.1007/s00330-026-12322-z)
Supplement: Supplementary file 1 — Supplementary Material [file 330_2026_12322_MOESM1_ESM.pdf]

A comprehensive multi-task deep learning model for kidney cancer: histological subtyping, clinical staging, and anatomical complexity grading

ELECTRONIC SUPPLEMENTARY MATERIAL

R.E.N.A.L. nephrometry score

The detailed scoring criteria refer to the study by Kutikov et al. and are provided in Supplementary Table 1 [1]. Tumor anatomical complexity grades were assessed by two experienced radiologists (G.Q., with 20 years of experience; W.C., with 30 years of experience) using the R.E.N.A.L. nephrometry score system. Any discrepancies between their initial readings were resolved through a consensus discussion, with a third senior radiologist serving as an arbiter for unresolved cases. Fifty patients were randomly selected for inter-rater reliability assessment between two radiologists. Reliability analyses were performed using the intraclass correlation coefficient (ICC) for continuous measurements and Cohen's kappa for nominal classifications. Supplementary Table 2 demonstrates good inter-observer agreement in assessing tumor anatomical complexity grades.

CT procedure

CT examinations were performed using six different scanner models, with detailed scanning parameters and device specifications provided in Supplementary Table 4. The scanning protocol comprised three phases: arterial phase (AP, immediately after contrast injection), venous phase (VP, 30-second delay post-injection), and delayed phase (DP, 90-second delay post-injection). A total of 50mL–65 mL of contrast agent (Ultravist, Bayer) was intravenously administered at an injection rate of 2.5 mL/s–3.0 mL/s, followed by a 30-mL saline flush.

ROI segmentation process

We anonymized the multiphase CT images. The AP, VP, and DP images were saved in NIFTI format. We obtained the dataset from the Kidney and Kidney Tumor Segmentation Challenge (KiTS23) (https://github.com/neheller/kits23) to train a 3D nnU-Net segmentation model for the preliminary segmentation of images from Center A and Center B (both as independent external test sets). A radiologist (T.L., 7 years of experience) independently reviewed these preliminary regions of interest (ROIs) and made corrections. Another radiologist (X.W., 10 years of experience) independently conducted the final review and modifications of the adjusted ROIs. The corrections made by both radiologists were completed using ITK-SNAP (version 3.8.0, http://www.itksnap.org). We used the Dice coefficient, 95% Hausdorff Distance (HD95) and Relative Volume Error (RVE) to evaluate the segmentation performance of the model (Supplementary Table 5).

This study observed a marked decline in segmentation model performance (e.g., elevated RVE) at the external validation Center B, clearly revealing domain shift issues between medical centers stemming primarily from differences in imaging equipment and acquisition protocols. Although radiologist-reviewed mask corrections ensured analytical quality, this finding highlights that enhancing the robustness of foundational segmentation models to cross-center domain shifts is equally as important as optimizing advanced classification models in the clinical translation of AI medical tools.

Sample size estimation

We estimated the sample size based on the area under the ROC curve (AUC). Among the 798 patients included in this study, we defined the positive groups as those with non-ccRCC, clinical stage III/IV, and high complexity in R.E.N.A.L. nephrometry scores, comprising 201 (25%), 150 (19%), and 187 (23%) cases, respectively. Using the "area under ROC curve" module in the "Sample size" tool of MedCalc application (version 20.0.4, https://www.medcalc.org), we set the Type I error at 0.05 and Type II error at 0.01, with the null hypothesis value set to 0.5. For the histologic subtype task, the AUC was set at 0.83 (referring to the result of Yang et al. [2]), with a negative-to-positive group ratio of 2.97. For the clinical staging task, the AUC was set at 0.80 (referring to the result of Demirjian et al. [3]), with a negative-to-positive group ratio of 4.32. For the R.E.N.A.L. score task, the AUC was set at 0.80, with a negative-to-positive group ratio of 3.27. The calculated sample sizes for all three tasks were smaller than our datasets, confirming that our datasets meet the sample size requirements (Supplementary Fig. 3).

Network details

Preprocessing of CT images and construction of the DataLoader were performed using the Medical Open Network for AI (MONAI, https://monai.io) deep learning framework. The pixel values of the images were normalized to the range [0, 1]. A 2-pixel margin was added around the tumor ROI for cropping, and both the images and their corresponding ROIs were resized to (32, 64, 64) to ensure consistent input dimensions for the network. Additionally, data augmentation techniques, including 90-degree rotation, random rotation within ± 10 degrees, and 3D elastic transformation, were applied to the training set to enhance the model's generalization capability.

The feature extraction network was initialized using a ResNet-34 pre-trained model from MedicalNet, accelerating convergence and enhancing network performance for 3D medical images. The 3D ResNet-34 backbone was employed to extract features from the ROIs of CT images in the arterial phase (AP), venous phase (VP), and delayed phase (DP). Following the feature extractor, a series of fully connected layers was used for further feature transformation and information processing. First, the input data was flattened using the nn.Flatten function to convert the multi-dimensional features of the triphasic images into a one-dimensional vector for subsequent processing. The data then underwent multiple linear transformations and nonlinear mappings via LeakyReLU activation functions. The feature dimension was projected to 1024 to provide an appropriate representation for the final classification task. To prevent overfitting, a dropout layer was added after the fully connected layers.

The final component of the model is a multi-task learning module that employs a Progressive Layered Extraction (PLE) network to handle three distinct tasks (including histological subtypes, clinical stages, and anatomical complexity grades). PLE is specifically designed to handle multiple tasks by explicitly distinguishing between shared and task-specific features to mitigate negative transfer issues [4]. The PLE architecture consists of three key components: ① an expert layer with separated shared experts (comprising num_shared_experts Deep Neural Networks (DNNs) for learning common features) and task-specific experts (num_task_experts per task for capturing unique features), all following the same expert_hidden_units configuration; ② a gating mechanism where each task's independent gate network dynamically computes expert combination weights through differentiable soft attention, enabling adaptive fusion of knowledge; and ③ task-specific towers where each task's prediction layer processes gated outputs through a DNN before mapping to label space via softmax for final probability distribution. This hierarchical structure effectively balances feature sharing and task specialization while minimizing interference between tasks. This study configured the model with 1 shared expert (num_shared_experts) and 3 task-specific experts (num_task_experts) for each task, with the hidden layer units (expert_hidden_units) in each expert module set to 256.

During the training of the multi-task model, we implemented a standard training iteration optimized for multi-task learning. To optimize each task in the multi-task learning framework, we employed the binary cross-entropy loss function (BCELoss), which calculates the discrepancy between the output of each task and its corresponding ground truth labels. The total loss across all three tasks was computed and used for backpropagation to update the network parameters, with equal weight ratios (1:1:1) assigned to each task's loss. The advantages of the equal-weighting method lie in: ① enabling fairer performance comparison between multi-task and single-task models compared to empirically tuned weights, and ② allowing the MTDL model to learn features that are meaningful for all three tasks, thereby preventing overfitting to any specific task. The following hyperparameters were configured for the training process: an initial learning rate (LR) of 0.0001, 200 training epochs, and a batch size of 32. The batch size was determined through a grid search under the memory constraints of our experimental hardware. Given that the model architecture efficiently performs feature-level fusion of triphasic images rather than simple channel stacking, this batch size achieves an optimal balance among training stability, memory utilization, and computational efficiency. We utilized the Adam optimizer to update the model parameters, leveraging its adaptive learning rate adjustment to accelerate training efficiency. Additionally, to enhance training stability, we incorporated a StepLR scheduler, which reduces the LR by a factor of 0.2 every 30 epochs. We have summarized the key parameters of the multi-task deep learning model in Table S6. The code for the multi-task deep learning model is available at https://github.com/clare-lyu/PLE-Multitask-training.

For single-task model training, each individual model adopted the same pre-trained backbone and training parameters as the multi-task model, followed by a linear classifier to construct three separate single-task models for their respective classification objectives. For a fair comparison, all three single-task models and the multi-task model were trained using the same data loader, ensuring they received identical batches of augmented data at each training step. All training procedures were conducted on a workstation with the following specifications: Intel i9-14900KF CPU, 2×32 GB DDR5 6000 RAM, and 2×NVIDIA RTX 4090 GPUs.

References

1. Kutikov A, Uzzo RG (2009) The R.E.N.A.L. nephrometry score: a comprehensive standardized system for quantitating renal tumor size, location and depth. J Urol 182: 844-853
2. Yang H, Liu H, Lin J et al (2024) An automatic texture feature analysis framework of renal tumor: surgical, pathological, and molecular evaluation based on multi-phase abdominal CT. Eur Radiol 34: 355-366
3. Demirjian NL, Varghese BA, Cen SY et al (2022) CT-based radiomics stratification of tumor grade and TNM stage of clear cell renal cell carcinoma. Eur Radiol 32: 2552-2563
4. Tang H, Liu J, Zhao M, Gong X (2020) Progressive Layered Extraction (PLE): A Novel Multi-Task Learning (MTL) Model for Personalized Recommendations: Proceedings of the 14th ACM Conference on Recommender Systems, Virtual Event, Brazil, 269-278

TABLE

Table S1 The detailed scoring criteria of the R.E.N.A.L. nephrometry score system

Scoring Criteria	Point		
	1pt	2pts	3pts
(R)adius (maximal diameter in cm)	≤4	>4 but <7	≥7
(E)xophytic/endophytic properties	≥50%	<50%	Entirely endophytic
(N)earness of the tumor to the collecting system or sinus (mm)	≥7	>4 but <7	≤4
(A)nterior/Posterior	No points given. Mass assigned a descriptor of a, p, or x		
(L)ocation relative to the polar lines	Entirely above the upper or below the lower polar line	Lesion crosses polar line	>50% of mass is across polar line or mass crosses the axial renal midline or mass is entirely between the polar lines
suffix "h" assigned if the tumor touches the main renal artery or vein			

Note: Scores of 4–6 represent low complexity, 7–9 represent intermediate complexity, and 10–12 represent high complexity.

Table S2 Consistency assessment of the R.E.N.A.L. nephrometry score in 50 random cases

Variables	ICC (95% CI)	*p* value
(R)adius (maximal diameter in cm)	0.996 (0.993~0.998)	<0.001
(N)earness of the tumor to the collecting system (mm)	0.995 (0.992~0.997)	<0.001
Variables	Kappa value (95% CI)	*p* value
(E)xophytic/endophytic properties	0.889 (0.767~1.000)	<0.001
(A)nterior/Posterior	0.933 (0.858~1.000)	<0.001
(L)ocation relative to the polar lines	0.910 (0.825~0.996)	<0.001
(h) touch the main renal artery or vein	0.935 (0.809~1.000)	<0.001
Grades (low / intermediate / high)	0.910 (0.809~1.000)	<0.001

ICC: intraclass correlation coefficient

Table S3 Baseline characteristics of the two centers

Characteristic	Total (n = 798)	Center A (n = 620)	Center B (n = 178)	*P* Value
Mean age (y)	54 ± 12	53 ± 13	58 ± 11	<0.001
Sex				
Female	279 (35.0)	218 (35.2)	61 (34.3)	0.826
Male	519 (65.0)	402 (62.8)	117 (65.7)	
BMI (kg/m²)	24.4 ±8.1	24.6 ± 9.0	23.6 ± 3.5	0.129
Maximum diameter	5.3 ± 2.9	5.3 ± 2.8	5.5 ± 3.1	0.433
Pathology type*				0.526
Clear cell RCC	597 (74.8)	463 (74.7)	134 (75.3)	
Papillary RCC	50 (6.3)	40 (6.5)	10 (5.6)	
Chromophobe RCC	81 (10.2)	65 (10.5)	16 (9.0)	
MiT family translocated RCC	10 (1.3)	7 (1.1)	3 (1.7)	
Other molecularly defined renal carcinomas	5 (0.6)	4 (0.6)	1 (0.6)	
Clear cell papillary renal cell tumor	5 (0.6)	2 (0.3)	3 (1.7)	
Eosinophilic solid and cystic RCC	5 (0.6)	5 (0.8)	0 (0)	
Mucinous tubular and spindle cell carcinoma	3 (0.4)	3 (0.5)	0 (0)	
Tubulocystic RCC	2 (0.3)	1 (0.2)	1 (0.6)	
Other malignant	40 (5.0)	30 (4.8)	10 (5.6)	

Note: Continuous variables were presented as mean ± standard deviation (SD), while categorical variables were expressed as counts and percentages. RCC = renal cell carcinoma. Independent samples t-test, chi-square test, or Fisher's exact test were employed to compare the baseline characteristics between the two centers.

* Pathology type grouping follows the WHO Classification of Tumors, 5th Edition, Volume 8: Urinary and Male Genital Tumors (tumourclassification.iarc.who.int).

Table S4 Details of the CT scanning parameters

Equipment	Center	Tube voltage (kV)	Tube current (mA)	Matrix	Slice thickness (mm)	Manufacturer
Revolution CT	A & B	100–120	200–500	512×512	5	GE Healthcare
uCT960+	A	120	350–500	512×512	3–5	United-Imaging
Brilliance iCT	A	100–120	100–350	512×512	5	Philips
Emotion 16	A	100–120	150–300	512×512	5	Siemens
Definition AS	A & B	100–120	120–400	512×512	3–5	Siemens
uCT760	B	120	150–300	512×512	5	United-Imaging

Table S5 The performance of the nnU-Net model in tumor segmentation

CT phase	Center A			Center B		
	Dice (normalized)	RVE (%)	HD95 (mm)	Dice (normalized)	RVE (%)	HD95 (mm)
Arterial phase	0.955 (0.939)	5.7	1.72	0.909 (0.908)	15.7	2.16
Venous phase	0.961 (0.946)	4.7	1.54	0.901 (0.901)	18.9	2.34
Delayed phase	0.960 (0.944)	5.4	1.51	0.902 (0.901)	18.1	2.70
Total	0.959 (0.943)	5.3	1.59	0.904 (0.903)	17.6	2.40

Note：The Dice coefficient measures the overlap between the predicted segmentation region (A) and the ground truth annotation region (B). Its calculation formula is Dice = 2×|A∩B| / (|A|+|B|), with a value range of 0 to 1. A value of 1 indicates perfect overlap, while 0 signifies no overlap at all. The Dice coefficient normalized to a fixed voxel-size of 1 mm^3 is reported in parentheses. The Relative Volume Error (RVE) quantifies the percentage difference between the predicted volume (V_pred) and the true volume (V_gt), calculated as RVE = |V_pred − V_gt| / V_gt ×100%. A value of 0% represents perfectly accurate volume prediction. The 95% Hausdorff Distance (HD95) is used to measure the maximum distance between two contours (typically the predicted segmentation region A and the ground truth annotation region B). Its calculation formula is HD (A, B) = max (h (A, B), h (B, A)).

Table S6 The key parameters of the multi-task deep learning model

Category	Parameter Name	Value/Description
Data Preprocessing	Pixel value normalization	[0, 1]
	Tumor ROI margin expansion	2 pixels
	Input image & ROI size	(32, 64, 64)
	Data augmentation methods	90° rotation,<br>±10° random rotation,<br>3D elastic transformation
Network Architecture	Feature extraction backbone	ResNet-34 (MedicalNet pre-trained)
	Fully connected layer output dimension	1024
	Activation function	LeakyReLU
	Regularization method	Dropout (rate = 0.3)
PLE Module	num_shared_experts	1
	num_task_experts	3 per task
	expert_hidden_units	256
Training Configuration	Loss function	Binary Cross-Entropy (BCELoss)
	Optimizer	Adam
	Initial learning rate (LR)	0.0001
	Training epochs	200
	Batch size	32
	Learning rate scheduler	StepLR (decay factor = 0.2 every 30 epochs)

Table S7 Comprehensive evaluation of multi-task and single-task deep learning performance

Metric	Task 1		Task 2		Task 3	
	MTDL	STDL	MTDL	STDL	MTDL	STDL
Validation set (n = 124)						
Precision (%)	95.9	97.8	52.9	44.2	51.5	48.7
Recall (%)	91.2	88.2	69.2	88.5	81.0	90.5
F1-score	0.94	0.93	0.60	0.59	0.63	0.63
Test set (n = 178)						
Precision (%)	91.0	91.2	56.9	42.3	57.7	62.0
Recall (%)	90.3	85.1	78.4	81.1	88.2	86.3
F1-score	0.91	0.88	0.66	0.56	0.70	0.72

Note: Precision and recall are expressed as percentages (%). The F1-score ranges from [0, 1], with values closer to 1 indicating better model performance. Precision = $\frac{TP}{TP+FP} \times 100\%$, Recall (Sensitivity) = $\frac{TP}{TP+FN} \times 100\%$, F1-score = $2 \times \frac{Precision \times Recall}{Precision + Recall}$, TP = true positives, FN = false negatives, FP = false positives. Task 1 is non-ccRCC vs. ccRCC, Task 2 is clinical stage I/II vs. III/IV, Task 3 is low-intermediate vs. high complexity. MTDL = multi-task deep learning. STDL = single-task deep learning.

Table S8 Pairwise correlation analysis of the three tasks within the overall cohort

Tasks	Clinical stage I/II	Clinical stage III/IV	χ²	*P*	Phi coefficient
ccRCC	500 (77.2)	97 (64.7)	10.090	0.002	-0.112
non-ccRCC	148 (22.8)	53 (35.3)			
Complexity low–intermediate	543 (83.8)	68 (45.3)	100.432	< 0.001	0.355
Complexity high	105 (16.2)	82 (54.7)			
Tasks	ccRCC	non-ccRCC	χ²	*P*	Phi coefficient
Complexity low–intermediate	142 (23.2)	469 (76.8)	5.247	0.027	-0.081
Complexity high	59 (31.6)	128 (68.4)			

Note: The chi-square test was employed, and all *P* values were corrected for continuity.

FIGURE

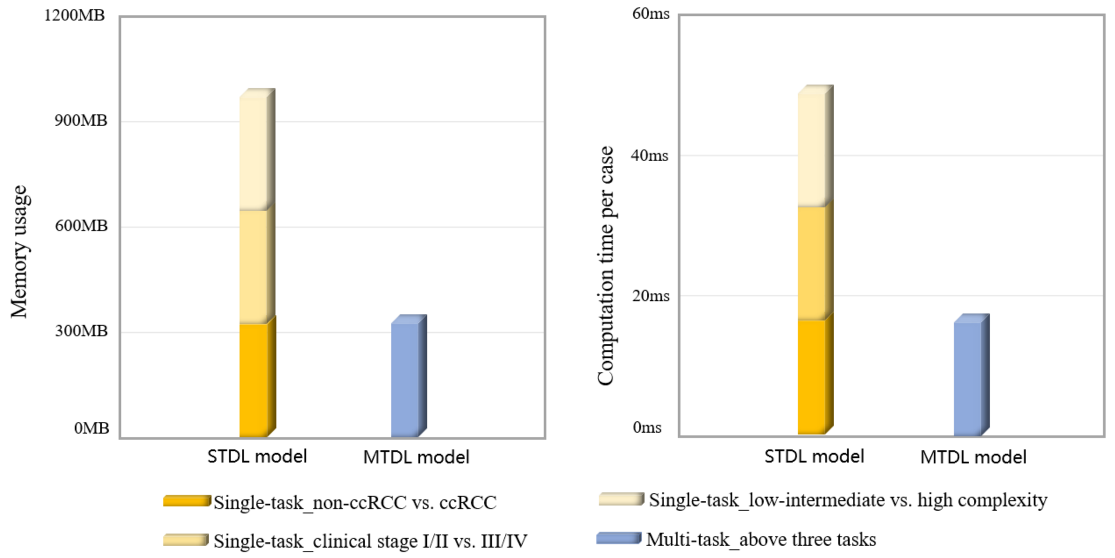

Fig. S1 Efficiency comparison between single-task deep learning (STDL) and multi-task deep learning (MTDL) algorithm models.

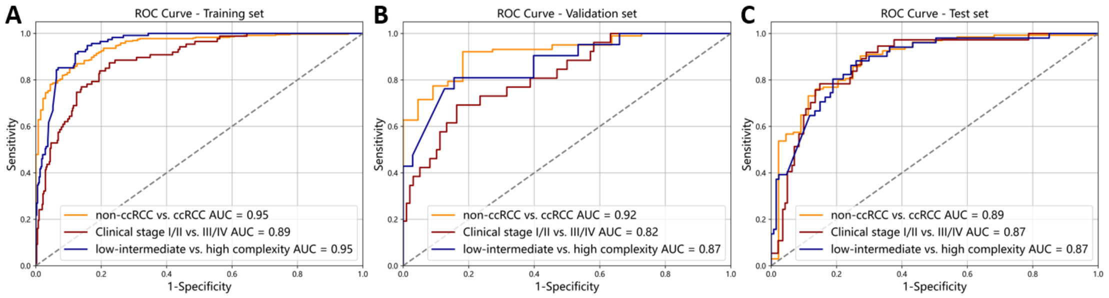

Fig. S2 Receiver operating characteristic curves of the multi-task deep learning algorithm model in the (A) training set, (B)internal validation set and (C) external test set.

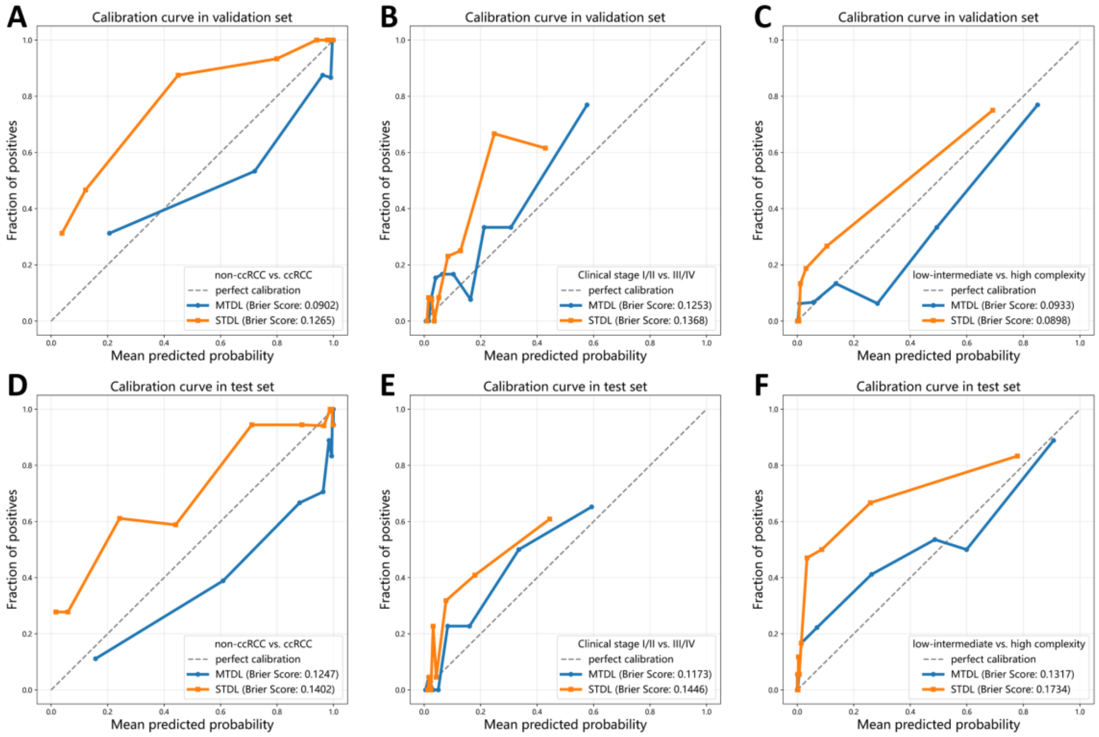

Fig. S3 Comparison of calibration curves between the multi-task deep learning algorithm model and single-task algorithm models in the (A–C) internal validation set and (D–F) external test set. The Brier Score is an evaluation metric used to assess the accuracy of probabilistic predictions. It quantifies a model's predictive performance by calculating the mean squared error between predicted probabilities and the actual observed outcomes. The Brier Score ranges from 0 to 1, with values closer to 0 indicating better predictive performance.

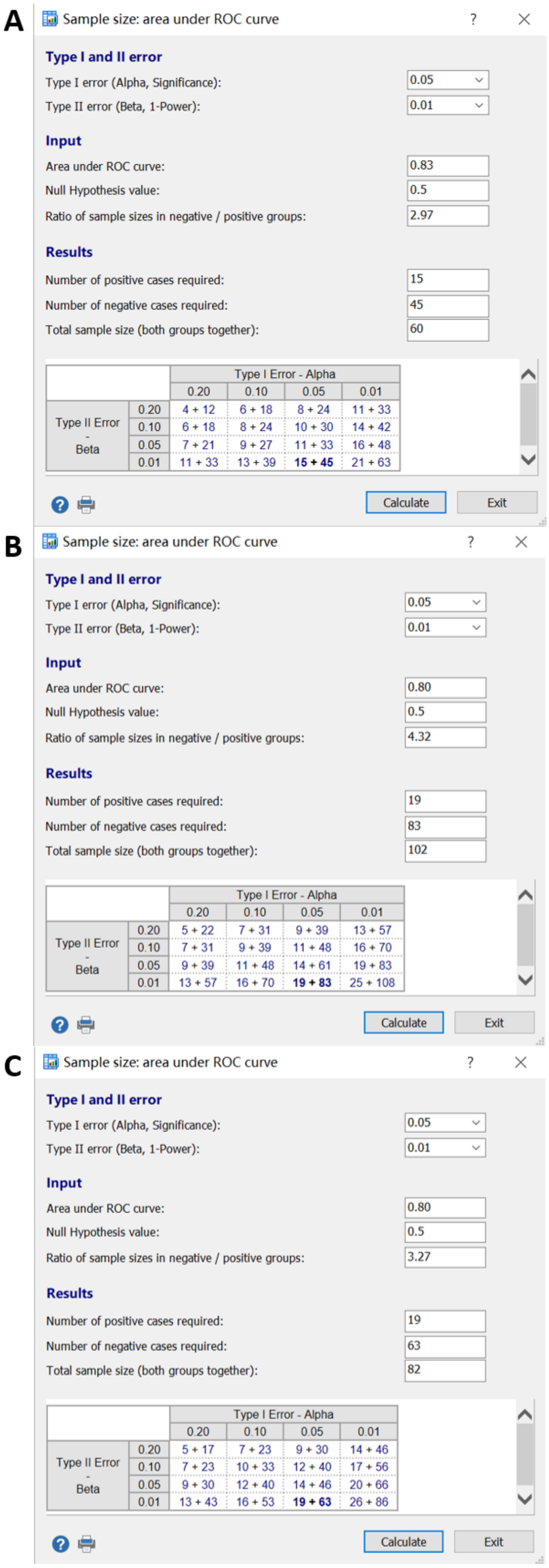

Fig. S4 Sample size estimation for the multi-task deep learning algorithm prediction model in identification of (A) histological subtypes, (B) clinical stages, and (C) anatomical complexity grades based on MedCalc application.
